# Relationship between plasma xanthine oxidoreductase activity and left ventricular ejection fraction and hypertrophy among cardiac patients

**DOI:** 10.1371/journal.pone.0182699

**Published:** 2017-08-10

**Authors:** Yuki Fujimura, Yohei Yamauchi, Takayo Murase, Takashi Nakamura, Shu-ichi Fujita, Tomohiro Fujisaka, Takahide Ito, Koichi Sohmiya, Masaaki Hoshiga, Nobukazu Ishizaka

**Affiliations:** 1 Department of Cardiology, Osaka Medical College, Osaka, Japan; 2 Mie Research Laboratories, Sanwa Kagaku Kenkyusho Co., Mie, Japan; Scuola Superiore Sant'Anna, ITALY

## Abstract

**Background and purpose:**

Xanthine oxidoreductase (XOR), which catalyzes purine catabolism, has two interconvertible forms, xanthine dehydrogenase and xanthine oxidase, the latter of which produces superoxide during uric acid (UA) synthesis. An association between plasma XOR activity and cardiovascular and renal outcomes has been previously suggested. We investigated the potential association between cardiac parameters and plasma XOR activity among cardiology patients.

**Methods and results:**

Plasma XOR activity was measured by [^13^C_2_,^15^N_2_]xanthine coupled with liquid chromatography/triplequadrupole mass spectrometry. Among 270 patients who were not taking UA-lowering drugs, XOR activity was associated with body mass index (BMI), alanine aminotransferase (ALT), HbA1c and renal function. Although XOR activity was not associated with serum UA overall, patients with chronic kidney disease (CKD), those with higher XOR activity had higher serum UA among patients without CKD. Compared with patients with the lowest XOR activity quartile, those with higher three XOR activity quartiles more frequently had left ventricular hypertrophy. In addition, plasma XOR activity showed a U-shaped association with low left ventricular ejection fraction (LVEF) and increased plasma B-type natriuretic peptide (BNP) levels, and these associations were independent of age, gender, BMI, ALT, HbA1C, serum UA, and CKD stages.

**Conclusions:**

Among cardiac patients, left ventricular hypertrophy, low LVEF, and increased BNP were significantly associated with plasma XOR activity independent of various confounding factors. Whether pharmaceutical modification of plasma XOR activity might inhibit cardiac remodeling and improve cardiovascular outcome should be investigated in future studies.

## Introduction

Individuals with higher serum uric acid levels are more likely to have cardiovascular risk factors, such as hypertension, diabetes, dyslipidemia, and obesity [1,2,3]. On the other hand, several cohort studies have demonstrated that hyperuricemia independently enhances cardiovascular risk [4,5], although the data are not always uniform [6,7], and gender difference remains a matter of debate [8]. On the other hand, treating hyperuricemia for the purpose of improving cardiovascular outcomes among asymptomatic patients is not currently internationally recommended, mainly due to a lack of placebo-controlled clinical trials studying the effects of urate-lowering therapy among such patients [9,10]. Of note, results of Mendelian randomization studies in which hyperuricemia played a causal role in cardiovascular outcome were also non-uniform [11,12,13,14]. Collectively these findings collectively raise the question of whether elevated circulating uric acid per se can causally enhance cardiovascular risk.

Uric acid is produced via the action of xanthine oxidoreductase (XOR), which catalyzes the last two steps of purine catabolism [15]. XOR has two interconvertible forms, xanthine dehydrogenase (XDH) and xanthine oxidase (XO). In contrast to XDH, which utilizes NAD^+^, XO utilizes O_2_ as an electron acceptor and generates superoxide during urate biosynthesis. It is thus possible that activation of XO, rather than uric acid, which has antioxidant capacity, may aggravate oxidant-induced cardiovascular injury [16,17]. Recently, we developed a novel and sensitive XOR assay for the measurement of human plasma XOR activity that is based on [^13^C_2_,^15^N_2_]xanthine coupled with liquid chromatography (LC)/triplequadrupole mass spectrometry (TQMS) [18,19].

To date, knowledge about the biomarker properties of plasma XOR activity among cardiac patients is limited. In the current study, therefore, we measured plasma XOR activity among patients with various cardiovascular disorders by LC–TQMS, and investigated whether plasma XOR activity is associated with laboratory or echocardiographic parameters.

## Methods

### Ethics statement

The current retrospective study was approved by the Ethics Committee at Osaka Medical College and conducted in accordance with the Declaration of Helsinki. Written informed consent was obtained from all patients or their guardians.

### Study population

**Fig 1:**
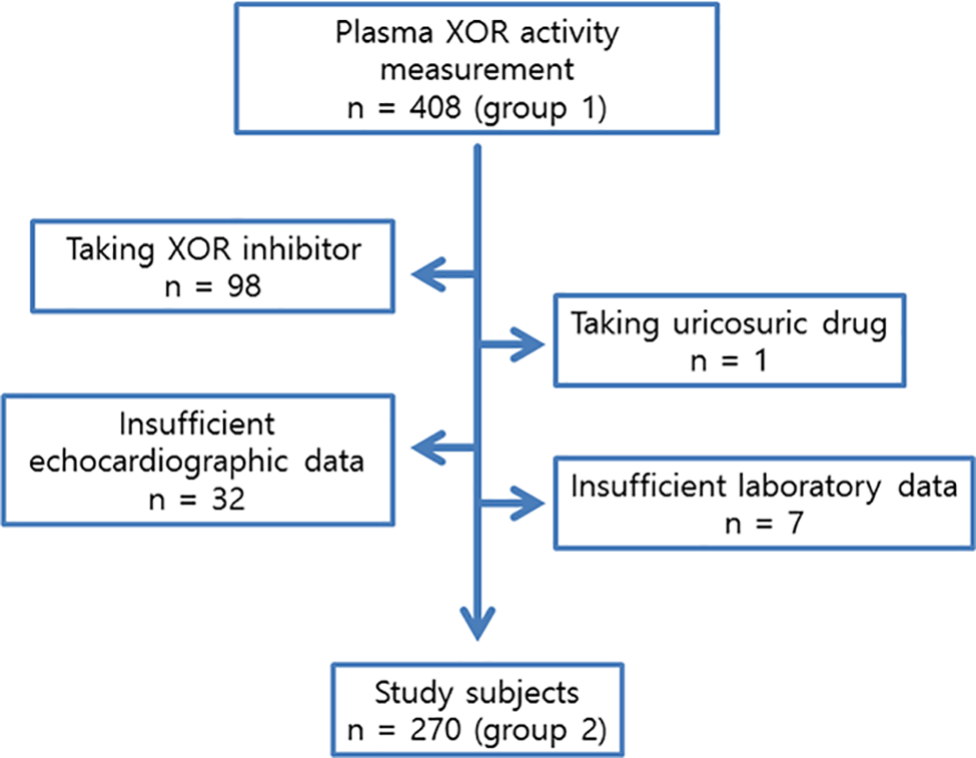
Flow diagram showing patient enrollment.

Among all patients admitted to the cardiology department between April 2016 and January 2014, plasma XOR activity was measured in 408, designated group 1, after obtaining written informed consent (Fig 1). Among the group 1 patients, 98 were taking an XOR inhibitory drug and excluded from the following study. In addition, 40 patients were excluded due to the administration of uricosuric drugs or insufficient echocardiographic or laboratory data. The remaining study population of 270 patients was designated group 2.

### Measurement of XOR activity

Aliquots of plasma were obtained and stored immediately at -80 degrees until analysis. Plasma XOR activity was determined by utilizing a combination of [^13^C_2_,^15^N_2_]xanthine and LT/QTMS as described.[19] In this method, the calibration curve showed linearity between 4 and 4000 nmol/L (R^2^> 0.995) with a lower limit of quantitation of 4 nmol/L, which corresponds to an XOR activity of 6.67 pmol/h/mL of plasma.

### Laboratory analysis

The estimated glomerular filtration rate (eGFR) was calculated by the following Modification of Diet in Renal Disease equation for Japanese subjects: eGFR = 194 × (serum creatinine)^-1.094^ × (age)^-0.287^ (× 0.739, when female) [20]. Renal function was graded as CKD stage G1 to G5 on the basis of eGFR level or requirement for hemodialysis [21], and the G3 category was further subdivided into early stage (G3a) and late stage (G3b) [22] as follows: G1 (eGFR > 90 mL/min/m^2^); G2 (eGFR 60–89 mL/min/m^2^); G3a (eGFR 45–59 mL/min/m^2^); G3b (eGFR 30–44 mL/min/m^2^); G4 (eGFR 15–29 mL/min/m^2^); and G5 (eGFR <15 mL/min/m^2^ or undergoing chronic hemodialysis). Patients with CKD stage 3b, 4, or 5 were considered to have moderate-to-severe renal failure, and those with CKD stages 3 or higher were considered to have CKD.

### Echocardiographic examination

Echocardiographic examinations was performed with a Vivid 7 Dimension instrument equipped with a multi-frequency transducer (GE Healthcare, Vingmed, Norway) as described.[23] In brief, left ventricular (LV) volumes were calculated by the modified Simpson method using the apical 4-chamber view and an LV ejection fraction (LVEF) of <50% was termed low LVEF. LV mass (LVM) was calculated by the formula proposed by Devereux et al. [24] with modification, and LVM index (LVMI) was calculated as the ratio of LVM to body surface area. LV hypertrophy (LVH) was defined to be present when the LVMI was greater than 118 (men)/108 (women) g/m^2^ [25].

### Statistical analysis

Baseline characteristics were assessed with standard descriptive statistics. Data were expressed as either mean±standard deviation or median (interquartile range). Spearman rank correlation test was used to assess the correlation between two variables. For the comparison of data among XOR activity quartiles, ANOVA, Mann-Whitney U test, or χ^2^ test was used. For multivariate analysis, multivariate linear regression and multivariate logistic regression analyses were used. Data analysis was performed by SPSS statistics version 22.0 (IBM, Armonk, NY). A value of P < 0.05 was taken to be statistically significant.

## Results

### XOR activity stratified by the XOR inhibitory drug administration in group 1 patients

**Fig 2:**
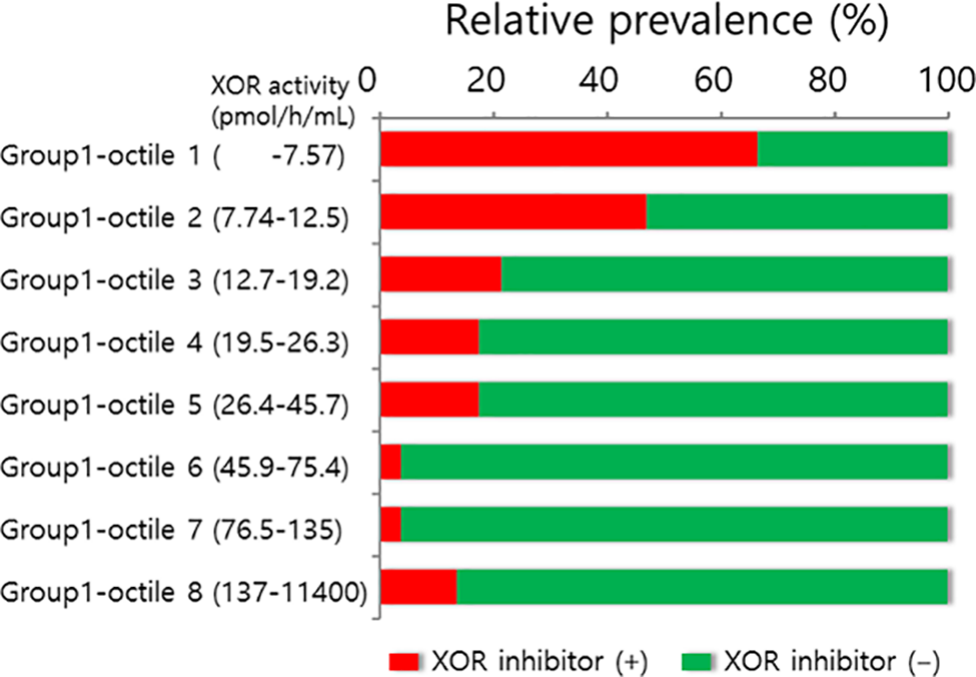
Percentage of group 1 patients who were and were not taking XOR inhibitory medication in each octile of plasma XOR activity.

Among 408 group 1 patients, 98 (24%) were taking an XOR inhibitory drug (allopurinol, febuxostat, or topiroxostat) (Fig 1). As compared with patients who were not taking XOR inhibitors, those treated by XOR inhibitors were significantly older (70.4 ± 11.1 years versus 74.7 ± 9.9, P = 0.001), had higher serum uric acid levels, and included a lower percentage of females (107 patients [34.5%] versus 16 [16.3%] P = 0.001). Plasma XOR activity was below the limit of quantification for 41 of the 408 patients. When the group 1 patients were subdivided by octile of XOR activity, those taking XOR inhibitors were significantly more prevalent in the lower octile value of XOR activity (Fig 2). Among 310 patients not taking XOR inhibitory drugs, only For 12 (3.9%) had plasma XOR activity below the lower limit of quantification.

### Clinical characteristics of group 2 patients stratified by plasma XOR activity

**Fig 3:**
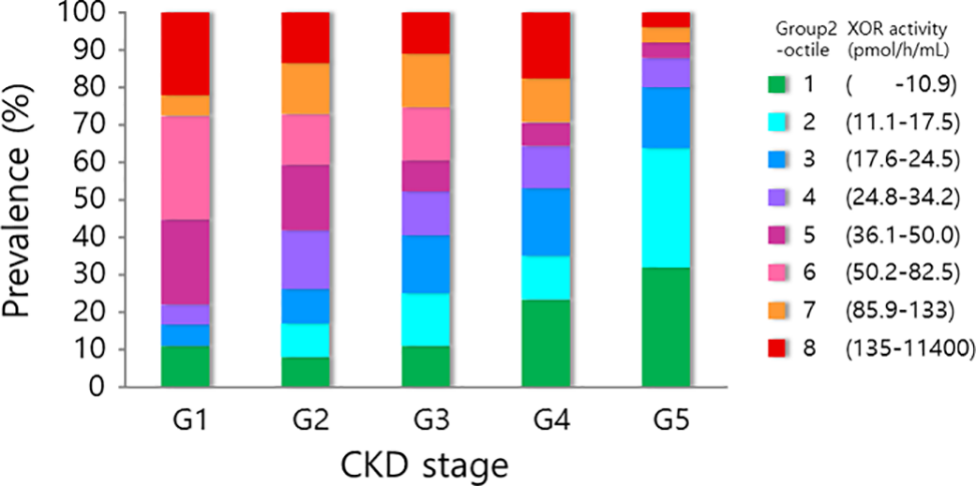
Distribution of plasma XOR activity octiles among group 2 patients with various stages of chronic kidney disease (CKD). Those who had worse renal function had significantly lower plasma XOR activity (P = 0.008, by χ^2^ test).

Next, we compared various clinical variables among group 2 patients, who were not taking urate lowering drugs, across quartiles of plasma XOR activity (Table 1). Those with higher XOR activity were younger and had greater body mass index (BMI), but gender prevalence did not differ significantly across the XOR quartiles. As compared with XOR activity below the median value, ever (i.e., former or current) smokers were more prevalent (70.1% versus 50.0%, P = 0.002) and moderate-to-severe renal dysfunction (i.e., CKD stages 3b, 4, and 5) was less prevalent (15.7% versus 42.6%, P<0.001) among those with XOR activity above the median value. When patients were subdivided by octile of XOR activity, those with relatively preserved renal function had relatively lower plasma XOR activity (P<0.001 by χ^2^ test, Fig 3).

**Table 1:** Clinical characteristics of group 2 patients by XOR activity quartile.

	XOR activity quartiles																
Variables	Q1 (n = 68)				Q2 (n = 68)				Q3 (n = 67)				Q4 (n = 67)				P value
XOR activity range, pmol/h/mL		-	17.5		17.6	-	34.2		36.1	-	82.5		82.9	-	11400		
women/men	27	/	41		28	/	40		15	/	52		21	/	46		0.078
Age, years	73.4	±	9.3		72.4	±	8.9		68.8	±	12.0		68.0	±	12.0		0.006
Body mass index, kg/m^2^	22.5	±	4.2		23.5	±	4.2		23.7	±	3.4		25.2	±	4.8		0.004
Systolic blood pressure, mmHg	133	±	24		121	±	21		129	±	23		125	±	22		0.014
NYHA III/IV, n (%)	20	(	29	)	23	(	34	)	7	(	10	)	23	(	34	)	0.005
*Smoking status*																	
Never, n (%)	37	(	54.4	)	31	(	45.6	)	19	(	28.4	)	21	(	31.3	)	0.022
Former, n (%)	27	(	39.7	)	33	(	48.5	)	40	(	59.7	)	36	(	53.7	)	
Current, n (%)	4	(	5.9	)	4	(	5.9	)	8	(	11.9	)	10	(	14.9	)	
CKD stages																	
G1	2	(	2.9	)	2	(	2.9	)	9	(	13.4	)	5	(	7.5	)	<0.001
G2	19	(	27.9	)	28	(	41.2	)	34	(	50.7	)	30	(	44.8	)	
G3a	13	(	19.1	)	14	(	20.6	)	14	(	20.9	)	21	(	31.3	)	
G3b	12	(	17.6	)	13	(	19.1	)	8	(	11.9	)	4	(	6.0	)	
G4	6	(	8.8	)	5	(	7.4	)	1	(	1.5	)	5	(	7.5	)	
G5	16	(	23.5	)	6	(	8.8	)	1	(	1.5	)	2	(	9.3	)	
*Cardiovascular disease*																	
Ischemic heart disease, n (%)	31	(	45.6	)	34	(	50.0	)	51	(	76.1	)	41	(	61.2	)	0.001
Arrhythmic disease, n (%)	23	(	33.8	)	33	(	48.5	)	21	(	31.3	)	28	(	41.8	)	0.155
Peripheral artery disease, n (%)	4	(	5.9	)	4	(	5.9	)	5	(	7.5	)	1	(	1.5	)	0.440
Valvular heart disease, n (%)	15	(	22.1	)	10	(	14.7	)	5	(	7.5	)	5	(	7.5	)	0.033
Cardiomyopathy, n (%)	9	(	13.2	)	8	(	11.8	)	3	(	4.5	)	7	(	10.4	)	0.346
Aneurysmal disease, n (%)	4	(	5.9	)	4	(	5.9	)	6	(	9.0	)	3	(	4.5	)	0.749
*Medication*																	
	32	(	47.1	)	23	(	33.8	)	38	(	56.7	)	32	(	47.8	)	0.064
Beta blockers, n (%)	20	(	29.4	)	25	(	36.8	)	20	(	29.9	)	30	(	44.8	)	0.203
Calcium channel blockers, n (%)	28	(	41.2	)	28	(	41.2	)	38	(	56.7	)	36	(	53.7	)	0.141
Any diabetic drug, n (%)	17	(	25.0	)	15	(	22.1	)	26	(	38.8	)	20	(	29.9	)	0.152
Statin, n (%)	24	(	35.3	)	29	(	42.6	)	32	(	47.8	)	25	(	37.3	)	0.452
Loop, n (%)	16	(	23.5	)	23	(	33.8	)	11	(	16.4	)	17	(	25.4	)	0.135
Thiazide, n (%)	16	(	23.5	)	23	(	33.8	)	11	(	16.4	)	17	(	25.4	)	0.282
Aldosterone antagonist, n (%)	7	(	10.3	)	13	(	19.1	)	6	(	9.0	)	8	(	11.9	)	0.285

### Laboratory and echocardiographic data stratified by plasma XOR activity among group 2 patients

Patients in the higher XOR activity quartiles had higher liver transaminase (alanine aminotransferase [ALT] and aspartate transaminase [AST]) levels (Table 2). HbA1c was significantly higher among those with higher XOR activity. Although serum uric acid did not differ significantly across the four XOR activity quartiles overall, patients without CKD and higher XOR activity had significantly higher serum uric acid levels

**Table 2:** Laboratory and echocardiographic data of group 2 patients by XOR activity quartile.

	XOR activity quartiles																								
Variables	Q1 (n = 68)						Q2 (n = 68)						Q3 (n = 67)						Q4 (n = 67)						P value
Laboratory data																									
White blood cell count, x10^3^/μL	5.7	(	4.6	-	7.3	)	5.4	(	4.2	-	7.0	)	6.0	(	4.9	-	7.1	)	5.7	(	4.5	-	7.1	)	0.654
Hemoglobin, g/dL	12.2	(	10.8	-	13.5	)	12.3	(	10.9	-	14.0	)	13.6	(	12.3	-	14.3	)	13.1	(	12.3	-	14.9	)	<0.001
Platelet count, x10^3^/μL	17.5	(	14.8	-	23.2	)	19.0	(	15.6	-	23.7	)	20.4	(	15.9	-	25.6	)	20.0	(	15.6	-	22.8	)	0.317
Total protein, mg/dL	6.9	(	6.4	-	7.3	)	6.8	(	6.3	-	7.1	)	7.0	(	6.5	-	7.4	)	6.9	(	6.5	-	7.3	)	0.303
Albumin, mg/dL	3.7	(	3.4	-	4.1	)	3.8	(	3.4	-	4.1	)	4.0	(	3.7	-	4.2	)	3.9	(	3.7	-	4.2	)	0.022
ALT. U/L	14	(	8	-	19	)	14	(	12	-	23	)	20	(	14	-	28	)	27	(	20	-	44	)	<0.001
AST. U/L	20	(	14	-	24	)	20	(	17	-	25	)	21	(	18	-	28	)	29	(	21	-	40	)	<0.001
Uric acid, mg/dL	5.5	(	4.0	-	7.3	)	5.3	(	4.4	-	7.0	)	5.8	(	4.7	-	6.9	)	6.4	(	5.2	-	7.9	)	0.094
Uric acid, mg/dL (eGFR≥60mL/min/m^2^)	4.4	(	3.9	-	5.4	)	4.8	(	4.2	-	5.5	)	5.3	(	4.6	-	6.3	)	6.0	(	4.1	-	6.9	)	0.007
Uric acid, mg/dL (eGFR<60mL/min/m^2^)	6.1	(	4.7	-	8.1	)	6.4	(	5.1	-	7.9	)	6.6	(	5.7	-	7.6	)	7.2	(	6.1	-	8.3	)	0.236
BNP, pg/mL	207	(	48	-	696	)	124	(	31	-	452	)	37	(	16	-	76	)	66	(	25	-	442	)	<0.001
Fasting glucose, mg/dL	104	(	92	-	139	)	111	(	92	-	135	)	111	(	95	-	153	)	112	(	97	-	145	)	0.443
HbA1c (NGSP), % (n = 251)	5.6	(	5.3	-	6.2	)	5.8	(	5.5	-	6.3	)	5.9	(	5.5	-	6.7	)	6.2	(	5.7	-	6.6	)	0.001
C-reactive protein, mg/dL	0.15	(	0.04	-	0.51	)	0.15	(	0.05	-	0.41	)	0.18	(	0.05	-	0.45	)	0.16	(	0.04	-	0.63	)	0.976
eGFR*, mL/min/1.73m^2^	53.6	(	36.5	-	65.1	)	56.2	(	41.1	-	69.5	)	67.0	(	52.9	-	81.0	)	62.1	(	50.5	-	72.1	)	0.001
Echocardiographic data																									
LVDd, cm	4.75	(	4.3	-	5.2	)	4.9	(	4.4	-	5.3	)	4.8	(	4.2	-	5.1	)	4.8	(	4.4	-	5.5	)	0.504
LVDs, cm	3.15	(	2.7	-	4.1	)	3.2	(	2.7	-	3.9	)	3.0	(	2.8	-	3.4	)	3.2	(	2.7	-	4.2	)	0.341
IVST, cm	1.0	(	0.9	-	1.1	)	0.9	(	0.8	-	1.0	)	1.0	(	0.8	-	1.1	)	0.9	(	0.8	-	1.1	)	0.026
PWT, cm	1.0	(	0.8	-	1.2	)	0.9	(	0.8	-	1.0	)	1.0	(	0.9	-	1.1	)	0.9	(	0.8	-	1.0	)	0.003
LV ejection fraction, %	58	(	46	-	69	)	61	(	50	-	69	)	63	(	57	-	70	)	61	(	44	-	67	)	0.096
LV mass index, g/m^2^	110	(	89	-	137	)	94	(	76	-	128	)	92	(	76	-	113	)	94	(	75	-	119	)	0.008

ALT, alanine aminotransferase; AST, aspartate aminotransferase; BNP, B-type natriuretic peptide; LVDd, left ventricular diastolic dimension; LVDs, left ventricular systolic dimension; IVST, intraventricular septal wall thickness; PWT, posterior wall thickness.

*For eGFR, those who were not undergoing chronic hemodialysis.

**Fig 4:**
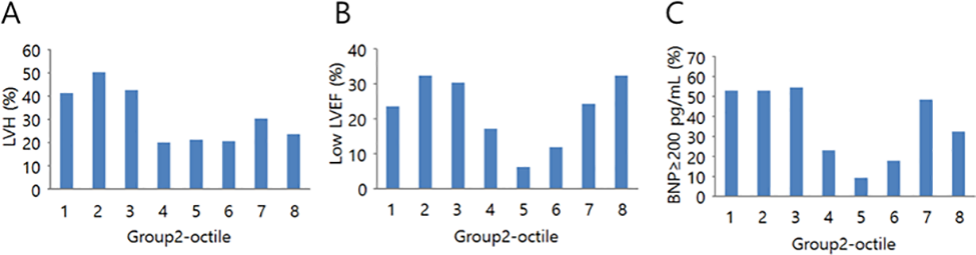
Percentage of group 2 patients with left ventricular hypertrophy (LVH), low left ventricular ejection fraction (LVEF), and elevated BNP (≥200 pg/mL) in each plasma XOR activity octile. A. Prevalence of LVH (P = 0.031, by χ^2^ test). B. Prevalence of low LVEF (P = 0.071). C. Prevalence of elevated BNP (P <0.001).

When stratified by XOR activity octile, the prevalence of LVH was higher in the first to third XOR activity octiles (Fig 4A); in addition, there was an apparent U-shaped association between XOR activity and both prevalence of low LVEF (Fig 4B) and elevated BNP (≥200 pg/mL) (Fig 4C).

### Multivariate logistic regression analysis

Next, the relationship between XOR activity quartiles, LVH, and low LVEF was examined by multivariate logistic regression analysis among group 2 patients (Table 3). As compared with the first XOR activity quartile, the third and fourth XOR activity quartiles were associated with LVH after adjusting for sex, age, and BMI (model 2). Increased prevalence of diastolic dysfunction remained significantly associated with the fourth XOR activity quartile after further adjustment for ALT, HbA1C, serum uric acid (model 3), and CKD stage (model 4).

**Table 3:** Logistic regression analysis for the association of XOR activity with left ventricular hypertrophy, low ejection fraction, and elevated BNP.

	Plasma XOR activity quartiles																							
	first						second						third						fourth					
	OR	(	95% CI			)	OR	(	95% CI			)	OR	(	95% CI			)	OR	(	95% CI			)
Dependent variable: left ventricular hypertrophy																								
Model 1	1	(	ref			)	0.53	(	0.26	-	1.08	)	0.32**	(	0.15	-	0.67	)	0.44**	(	0.21	-	0.90	)
Model 2	1	(	ref			)	0.51	(	0.25	-	1.04	)	0.29**	(	0.13	-	0.63	)	0.38**	(	0.18	-	0.81	)
Model 3	1	(	ref			)	0.38	(	0.18	-	0.84	)	0.22**	(	0.10	-	0.51	)	0.24*	(	0.10	-	0.58	)
Model 4	1	(	ref			)	0.47	(	0.21	-	1.06	)	0.33*	(	0.14	-	0.80	)	0.34**	(	0.14	-	0.86	)
Model 5	1	(	ref			)	0.51	(	0.22	-	1.22	)	0.40*	(	0.16	-	0.99	)	0.45	(	0.18	-	1.17	)
Dependent variable: low left ventricular ejection fraction																								
Model 1	3.94**	(	1.46	-	10.6	)	3.13*	(	1.14	-	8.58	)	1.00	(	ref			)	4.02**	(	1.49	-	10.9	)
Model 2	4.21**	(	1.54	-	11.5	)	3.27*	(	1.18	-	9.07	)	1.00	(	ref			)	3.94**	(	1.45	-	10.7	)
Model 3	5.32**	(	1.72	-	16.5	)	3.70*	(	1.20	-	11.4	)	1.00	(	ref			)	3.02	(	0.98	-	9.29	)
Model 4	3.93*	(	1.20	-	12.8	)	3.10	(	0.99	-	9.77	)	1.00	(	ref			)	2.64	(	0.84	-	8.27	)
Model 5	3.85*	(	1.14	-	13.0	)	3.02	(	0.93	-	9.74	)	1.00	(	ref			)	3.06	(	0.98	-	9.53	)
BNP ≥200 pg/mL																								
Model 1	7.25**	(	3.10	-	16.9	)	3.99**	(	1.70	-	9.4	)	1.00	(	ref			)	4.35**	(	1.85	-	10.2	)
Model 2	6.15**	(	2.58	-	14.7	)	3.38**	(	1.41	-	8.1	)	1.00	(	ref			)	4.68**	(	1.93	-	11.4	)
Model 3	8.67**	(	3.26	-	23.1	)	3.16*	(	1.22	-	8.2	)	1.00	(	ref			)	2.86*	(	1.08	-	7.55	)
Model 4	4.08*	(	1.36	-	12.3	)	2.67	(	0.94	-	7.6	)	1.00	(	ref			)	2.67	(	0.93	-	7.67	)
Model 5	4.57*	(	1.34	-	15.6	)	2.47	(	0.78	-	7.9	)	1.00	(	ref			)	3.41*	(	1.05	-	11.0	)

*indicates P<0.05

** indicates P<0.01.

To assess of the relationship between XOR activity quartile and low LVEF, the third XOR activity quartile was used as a reference. As a result, the first, second, and fourth XOR activity quartiles were associated with low LVEF after adjusting for sex, age, and BMI (model 2). Increased prevalence of low LVEF remained significantly associated with the first and second XOR activity quartiles after further adjustment for ALT, HbA1C, and serum uric acid (model 3). The U-shaped relationship between the XOR activity and elevated BNP remained significant even further adjustment for CKD stage and diuretic use (model 5).

## Discussion

In the current study, we demonstrated that plasma XOR activity was associated BMI, liver enzymes, and HbA1c. In addition, plasma XOR activity was associated negatively with renal function and cardiac hypertrophy, and showed a U-shaped association with low LVEF and elevated plasma BNP; these associations were independent of BNP, ALT, HbA1c, uric acid, CKD stages, and diuretic use.

When the patients were subdivided according to the presence or absence of CKD, higher XOR levels were significantly associated with higher uric acid only among those with preserved renal function, in agreement with a previous observation [26]. This may be because serum uric acid levels are more influenced by the uric acid production among subjects with preserved renal function than among those with CKD, in which serum uric acid increases owing to decreased urate excretion. Nevertheless, because serum uric acid is affected by various parameters including diuretic use that can affect urinary urate excretion [27,28], this possibility should be re-assessed in future studies based on larger study population.

The organs and/or tissues from which plasma XOR originates in cardiac patients are not clear. On the other hand, it has been presumed that hepatic XOR will be released into systemic circulation upon certain noxious stimuli, such as hyperglycemic conditions, and this may aggravate vascular function impairment [29]. Rootwelt demonstrated that the release of XOR into the circulation from injured tissues after hypoxia and subsequent re-oxygenation [30]. XOR is shown to appear in the systemic circulation after ischemia reperfusion in humans [31]. On the other hand, XOR may also bind to the vascular endothelium, which might inhibit nitric oxide-dependent cGMP production in a superoxide dismutase-resistant manner [32]. Nakamura et al. reported that increased circulating XOR activity is related along with the increase in urinary albumin excretion in diabetic mice [33]. Although several studies have suggested that circulating XOR may have physiological importance, or at least biomarker properties in animal models, determination of human plasma XOR activity had, in general, been difficult owing to its low levels [34].

We recently developed a sensitive XOR assay for the measurement of human plasma XOR activity by utilizing [^13^C_2_,^15^N_2_]xanthine coupled with LC–TQMS.[18,19] Via this novel method, Otaki et al. recently showed that patients with congestive heart failure who had high plasma XOR activity had significantly lower LVEF, and that those with low, or high XOR activity had increased cardiovascular events and reduced survival rate [26]. The mechanism underlying the U-shaped association between plasma XOR activity and cardiovascular outcome remains unknown; however, it has been speculated that patients with low XOR activity might be om a relatively cachexic condition and thus susceptible to reduced synthesis proteins including XOR [35]. In the study population (group 2), on the other hand, the median value of serum total protein in both the lowest and the highest XOR quartile was to 6.9 mg/dL, suggesting that this U-shape association might not be explained by the cachexia. In agreement with Otaki et al.’s findings, those with low XOR activity had advanced age and low eGFR, which might affect the prevalence of cardiac dysfunction and plasma BNP levels. Nevertheless, in the current study, the U-shape association between XOR activity and low LVEF or elevated BNP was suggested to be independent of various possible confounding variables, including age, CKD stage, and diuretic use.

By measuring the XO-specific conversion of lumazine to isoxantholumazine, Tam et al. showed that obese children had highly elevated XO activity as compared with their healthy weight counterparts, and that XO activity correlated positively with BMI z-score, waist circumference, and oxidized low-density lipoprotein, and negatively with high-density lipoprotein cholesterol [36]. We also found that patients with higher XOR activity had higher BMI. The finding that weight loss was associated with the decreased XO activity further support the notion that being overweight and/or obese might influence circulating XOR activity [37,38]. We also found that patients with higher plasma XOR activity had higher HbA1C levels. Miric et al. reported that serum XO activity was higher among type 2 diabetic patients than among non-diabetic control subjects, and that serum XO activity was directly correlated with BMI in patients with diabetes [39]. Our current finding that plasma XOR activity was significantly associated with ALT and AST is in agreement with previous findings in human studies [19]. Considering that those who had higher XOR activity had higher liver enzymes, the potential hepatocardiac relationship from the viewpoint of XOR activity should be investigated in future studies.

The current study has a number of limitations. First, NAD^+^ was included in the reaction mixture for the measurement of XOR activity; therefore, the assay measured theoretically both XDH and XO activity [19]. Second, the patients enrolled in the current study were taking various medications that might affect plasma XOR activity [40]; however, we could not take these differences into consideration owing to the relatively small sample size. Third, because XOR has affinity for heparin and can bind to vascular endothelial cells [41], administration of heparin before blood collection might increase XOR activity in the circulating blood; however, heparin injection was not performed before blood sampling in accordance with other investigators’ methods of XOR activity measurement [17,26,34], and also to avoid potential adverse effects [42].

In conclusion, we found that patients with higher three XOR activity quartiles had lower prevalence of LVH compared with those with the lowest XOR activity quartile. On the other hand, plasma XOR activity showed a U-shaped association with low LVEF and elevated BNP, independent of other confounding factors, including age, gender, BMI, ALT, serum uric acid, HbA1C, renal function, and diuretic use. Whether pharmaceutical modification of plasma XOR activity might retard cardiac remodeling and improve cardiovascular outcome should be investigated in future studies.

## Abbreviations

XOR: Xanthine oxidoreductase
BMI: uric acid
ALT: alanine aminotransferase
CKD: chronic kidney disease
XDH: xanthine dehydrogenase
XO: xanthine oxidase
LC: liquid chromatography
TQMS: triplequadrupole mass spectrometry
eGFR: estimated glomerular filtration rate
LV: left ventricular
LVM: left ventricular mass
LVMI: left ventricular mass index
LVH: left ventricular hypertrophy
AST: aspartate transaminase
BNP: B-type natriuretic peptide
